# Biochemical characterization of *Escherichia coli* DnaC variants that alter DnaB helicase loading onto DNA

**DOI:** 10.1016/j.jbc.2024.107275

**Published:** 2024-04-06

**Authors:** Sarah D. McMillan, James L. Keck

**Affiliations:** Department of Biomolecular Chemistry, University of Wisconsin, Madison, Wisconsin, USA

**Keywords:** ATPase, AAA+ ATPase, DNA, DnaB, DNA helicase, DnaC, DNA helicase loader, DNA replication, DNA replication restart

## Abstract

DNA replication in *Escherichia coli* starts with loading of the replicative helicase, DnaB, onto DNA. This reaction requires the DnaC loader protein, which forms a 6:6 complex with DnaB and opens a channel in the DnaB hexamer through which single-stranded DNA is thought to pass. During replication, replisomes frequently encounter DNA damage and nucleoprotein complexes that can lead to replication fork collapse. Such events require DnaB re-loading onto DNA to allow replication to continue. Replication restart proteins mediate this process by recruiting DnaB_6_/DnaC_6_ to abandoned DNA replication forks. Several *dnaC* mutations that bypass the requirement for replication restart proteins or that block replication restart have been identified in *E. coli*. To better understand how these DnaC variants function, we have purified and characterized the protein products of several such alleles. Unlike wild-type DnaC, three of the variants (DnaC 809, DnaC 809,820, and DnaC 811) can load DnaB onto replication forks bound by single-stranded DNA-binding protein. DnaC 809 can also load DnaB onto double-stranded DNA. These results suggest that structural changes in the variant DnaB_6_/DnaC_6_ complexes expand the range of DNA substrates that can be used for DnaB loading, obviating the need for the existing replication restart pathways. The protein product of *dnaC1331*, which phenocopies deletion of the *priB* replication restart gene, blocks loading through the major restart pathway *in vitro*. Overall, the results of our study highlight the utility of bacterial DnaC variants as tools for probing the regulatory mechanisms that govern replicative helicase loading.

In bacteria, DNA replication begins with DnaA binding to and melting the origin of replication (1, 2). DnaA then recruits the replicative helicase (DnaB in Gram-negative bacteria) to the origin and DnaB is loaded onto each strand of the single-stranded DNA (ssDNA) bubble (3). DnaB exists as a closed ring-shaped hexamer and ssDNA must bind in the interior of the ring (4, 5). This topological challenge is overcome by a loading factor, DnaC in *Escherichia coli*, that binds to DnaB and promotes loading (6, 7, 8).

DnaC binding to DnaB causes a large structural rearrangement of the helicase that is thought to be important for loading onto ssDNA. DnaC is comprised of an N-terminal DnaB helicase-binding domain and a C-terminal AAA+ ATPase domain (Fig. 1*A*) (7). DnaC assembles onto DnaB to form a DnaB_6_/DnaC_6_ complex (Fig. 1*B*) (9). Upon DnaC binding, DnaB’s C-terminal helicase domains adopt a spiral shape within the hexamer, breaking the planar arrangement observed in apo-DnaB structures whereas DnaB’s N-terminal domains lock into a constricted conformation (9). Importantly, DnaC binding also produces an opening, or crack, in the DnaB hexamer that is thought to provide an entry point for ssDNA to load into the interior of the hexameric helicase. After ssDNA loading, DnaC hydrolyzes ATP, triggering DnaC dissociation and closure of the DnaB hexamer around ssDNA (9, 10). DnaB is then competent to unwind parental DNA and recruit other components of the cellular replication machinery (replisome), which begin bidirectional replication around the chromosome (11, 12, 13).

**Figure 1:**
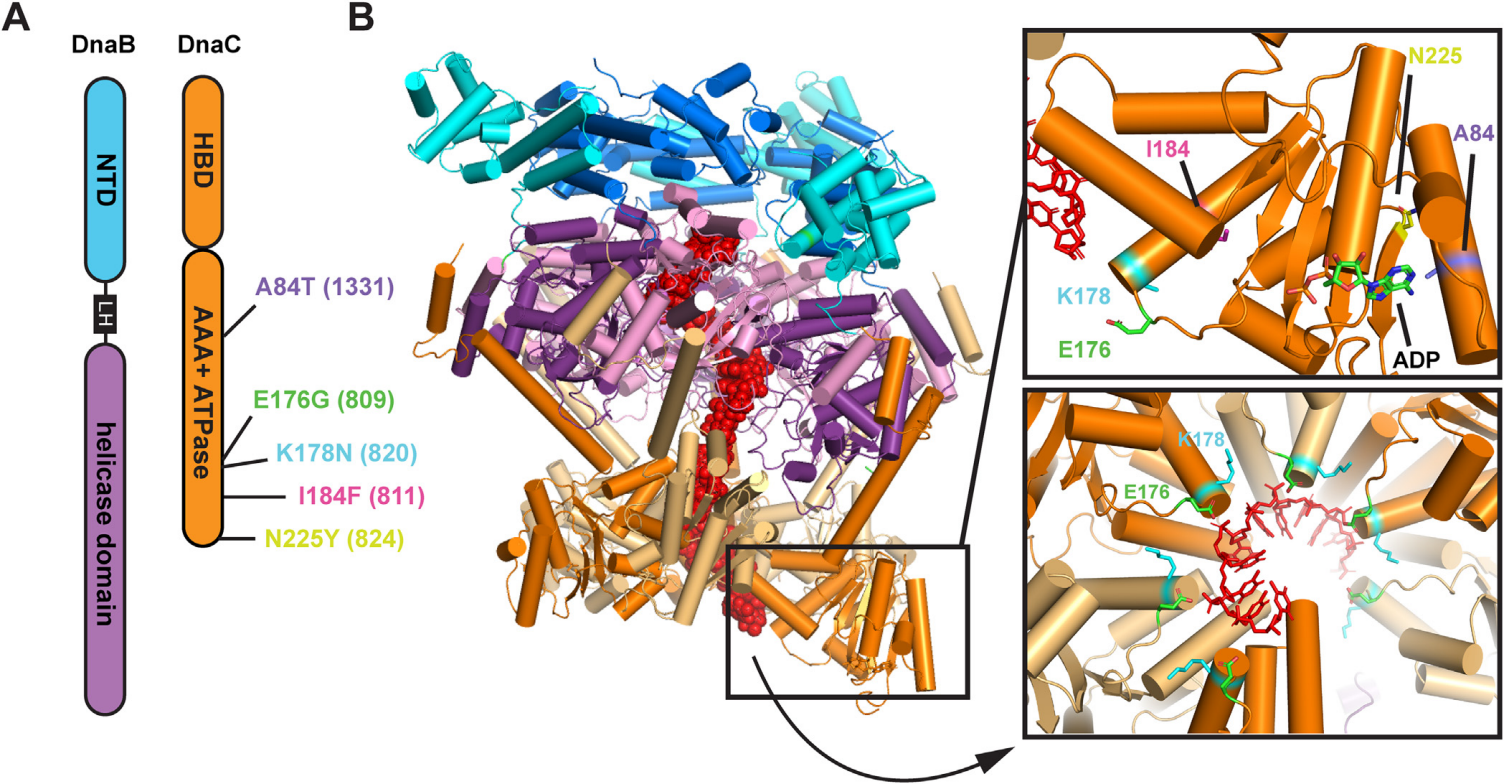
**DnaC mutations map to its AAA+ ATPase domain.***A*, domain layout of DnaB and DnaC. DnaB contains an N-terminal domain (NTD, *blue*) and a C-terminal helicase domain (*purple*) connected by a linker helix (LH). DnaC contains an N-terminal helicase-binding domain (HBD) and a AAA+ ATPase domain. *B*, structure of DnaB_6_/DnaC_6_ bound to ssDNA (9). The DnaC variant sites examined here are mapped onto the AAA+ ATPase domain (*insets*).

Replisomes routinely encounter impediments such as tightly bound nucleoprotein complexes or damaged DNA that can stall replication or, in severe cases, prematurely eject the replisome from DNA (14, 15). The latter events are frequent in bacteria—it is estimated that each replisome in *E. coli* synthesizes only a fraction of the chromosome before encountering an impediment that causes it to dissociate (15, 16, 17, 18). Because DnaA loads DnaB in a sequence-dependent manner at the origin of replication, these occurrences require reloading of DnaB by a distinct process mediated by replication restart proteins (RRPs, PriA, PriB, PriC, and DnaT in *E. coli*) (16). RRPs bind replication forks in a structure-specific manner, remodel the replication fork to expose lagging-strand ssDNA, and recruit DnaB_6_/DnaC_6_ to load DnaB onto the lagging strand. Remodeling occurs either through unwinding of the lagging strand when it is duplex or exposure of ssDNA from ssDNA-binding proteins (SSBs) that coat lagging-strand ssDNA (17). The mechanisms by which RRPs recruit and load the DnaB_6_/DnaC_6_ complex are not well understood.

In *E. coli*, there are three genetically defined replication restart pathways (18). Two utilize PriA and either PriB or PriC. A third PriA-independent pathway utilizes PriC and the Rep helicase. The PriA/PriB restart pathway is thought to be the most heavily utilized in cells, and PriA-deficient cells exhibit severe phenotypes including slow growth, rich media sensitivity, SOS induction, filamentation, and ultraviolet light sensitivity (19, 20, 21, 22).

Genetic studies have identified several mutations that suppress replication restart defects in *E. coli*. Intriguingly, these suppressors are missense mutations that map to *dnaC* (Fig. 1), suggesting that the mutations produce DnaC variants that can bypass the need for RRPs to load DnaB *in vivo* (22, 23). Understanding how such DnaC variants function could provide insights into the general DnaB loading process.

The protein product of one Δ*priA* suppressor, *dnaC809*, has been studied previously. Unlike wild-type DnaC, DnaC 809 can load DnaB onto SSB-bound ssDNA without the assistance of RRPs (24). The biochemical activities of additional Δ*priA* bypass mutant protein products, including DnaC 809,820 (suppresses a Δ*priA* Δ*priC* mutant lacking all replication restart pathways) and DnaC 811 (suppresses Δ*priA* similarly to *dnaC809*) (22, 23), have not been examined, making it unclear whether observations made with DnaC 809 explain the functions of all *dnaC* bypass mutants.

In addition to Δ*priA* suppressors, other *dnaC* alleles have been isolated that suppress or mimic mutations in different RRP genes (25). The *dnaC824* allele suppresses the filamentation and SOS induction phenotypes observed in Δ*priB* Δ*rep* cells but does not suppress Δ*priA* phenotypes to the same extent as *dnaC* bypass alleles found in the Δ*priA* background. Alternatively, *dnaC1331* is a loss of function allele that phenocopies deletion of the *priB* gene *in vivo*. Although a *priB* deletion on its own does not exhibit severe phenotypes, it cannot be combined with a *priC* deletion (18). The biochemical consequences of the *dnaC1331* mutation have not been investigated.

In this study, we took a biochemical approach to examine the impact of *dnaC* mutations in five DnaC variants: DnaC 809 (E176G), DnaC 809,820 (E176G, K178N), DnaC 811 (I184F), DnaC 824 (N255Y) and DnaC 1331 (A84T). The first four variants are the products of *dnaC* missense mutations that suppress a variety of replication restart-deficient backgrounds whereas the fifth, DnaC 1331, interferes with normal replication restart function *in vivo*. All five variants were purified and shown to retain the ability to bind DnaB and ssDNA, which was expected given that they all function *in vivo*. Each variant was also able to load DnaB onto a synthetic replication fork with an ssDNA lagging strand in the absence of SSB. Three variants, DnaC 809, DnaC 809,820, and DnaC 824, were found to also load DnaB onto SSB-coated replication forks, an activity that is not supported by wild-type DnaC. DnaC 809 was unique among the variants in displaying an unexpected ability to load DnaB onto replication forks with duplex lagging strand DNA. While DnaB has been shown to translocate across duplex DNA previously (26, 27), its loading onto duplex DNA has not been observed. Finally, DnaC 1331 blocked the loading of DnaB through the PriA-PriB replication restart pathway *in vitro*, consistent with its ability to phenocopy *priB* deletion *in vivo*. The results collectively suggest that *dnaC* suppressor bypass functions can arise from DnaC variant loading of DnaB onto a broader spectrum of substrates than is supported by wild-type DnaC and provide new insights into the connections between RRPs and DnaB loading.

## Results

### DnaC variants retain the ability to bind and load DnaB onto a replication fork that does not require remodeling

We took a biochemical approach to examine how sequence changes in DnaC can either allow cells to bypass the need for RRPs to load DnaB onto DNA or inhibit replication restart. Four DnaC variants that suppress a variety of replication restart deficient backgrounds were purified: DnaC 809 (E176G), DnaC 809,820 (E176G, K178N), DnaC 811 (I184F), and DnaC 824 (N225Y). Additionally, DnaC 1331 (A84T), which mimics *priB* deletion, was purified. Each variant carried one or two substitutions in the C-terminal AAA+ domain of DnaC (Fig. 1).

**Figure 2:**
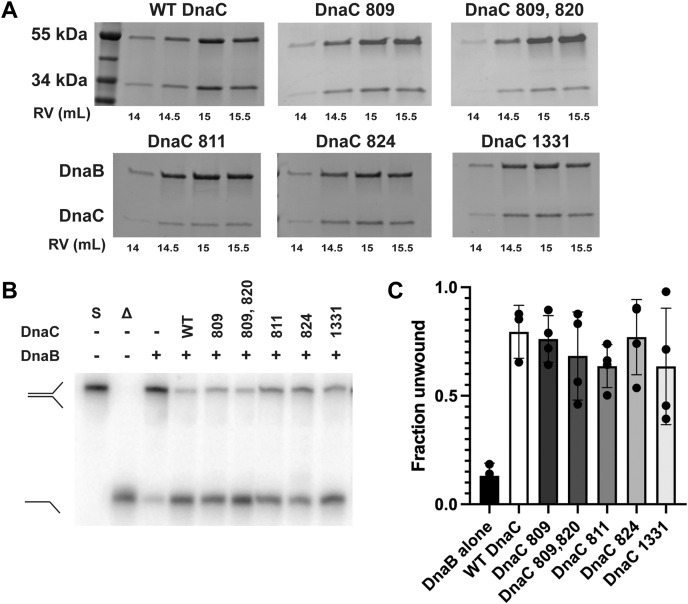
**DnaC variants retain the ability to bind DnaB and load DnaB onto free ssDNA.***A*, SDS-PAGE of peak fractions from analytical size exclusion chromatography with DnaB and each DnaC variant. Retention volumes (RTs) are reported below each gel. *B*, helicase loading assay with a replication fork containing 60 bp duplex parental DNA and 38 base ssDNA leading- and lagging-strand arms. Formation of unwound product indicates DnaB has been loaded and unwinds the parental duplex. Unwinding was measured three or more times, with the gel representative of multiple experiments. S = substrate and Δ = boiled substrate. *C*, bars display the mean fraction of DNA unwound from three or more independent experiments, with the values for each measurement shown as points. Error bars represent standard error.

The variants were first tested for their ability to bind to DnaB and to ssDNA. We predicted that since each of the variants functions in cells, they would retain interactions with DnaB and DNA. Purified DnaC variants were combined with DnaB in a 1.2:1 (DnaC:DnaB) ratio and complexes were resolved *via* size exclusion chromatography. Consistent with our prediction, each of the DnaC variants coeluted with DnaB, indicating that they interacted with the helicase (Figs. 2*A* and S1). Some of the DnaB/DnaC variant peaks were shifted to slightly higher retention volumes or to lower peak heights, potentially indicating that these variants dissociate more readily than wild-type DnaC during the non-equilibrium size exclusion experiment or that the stoichiometry is different for some variants. The ssDNA binding activity of each variant was also very similar to wild-type DnaC, with most variants binding as well as wild-type DNA and only DnaC811 displaying slightly weakened binding (Fig. S2). Thus, the DnaC variants retained interactions with DnaB and DNA, indicating that the sequence changes did not significantly alter folding.

We next measured the abilities of the DnaC variants to load DnaB onto radiolabeled synthetic DNA replication forks. The first substrate examined was the simplest possible replication fork structure with a parental DNA duplex and single-stranded leading and lagging strands that are free from SSB (Fig. 2, *B* and *C*, Table S1). Loading activity was assessed by measuring DnaB-dependent unwinding of parental DNA in the replication fork, which is observed *via* gel electrophoresis. We predicted that each DnaC variant would retain the ability to load DnaB onto this simple replication fork since each can load DnaB in cells. As reported previously (28), DnaB alone unwinds only a small fraction (∼10%) of the substrate, indicating that the protein could load onto a free replication fork with limited efficiency. The inclusion of wild-type DnaC increased DnaB unwinding efficiency to ∼80%, consistent with its known role as a loader protein for DnaB. Each of the five DnaC variants also stimulated DnaB loading, producing similar levels of unwound product (60–80%) to that observed with wild-type DnaC. Therefore, each of the DnaC variants retained DnaB and ssDNA binding and was able to load DnaB onto a simple DNA replication fork.

### Several DnaC bypass variants can load DnaB onto replication fork structures with single-stranded lagging strands in the presence of SSB

We next examined whether the DnaC bypass variants could overcome the need for RRPs *in vitro* as suggested by their abilities *in vivo* (18, 22, 25). Two scenarios can prevent DnaC-mediated DnaB loading onto lagging stands in cells. When the lagging strand is single-stranded, SSB coats the strand and prevents DnaB loading (24, 29). Alternatively, when the lagging strand is duplex, DnaC is blocked from loading DnaB (26). DNA replication restart pathways can remodel either substrate to allow DnaC-dependent DnaB loading in these situations, and the reactions have been reconstituted with synthetic replication forks *in vitro* (29, 30). The RRP suppressor phenotypes of *dnaC809*, *dnaC809,820*, *dnaC811*, and *dnaC824* suggest that the variants encoded by these alleles may be able to overcome challenges in loading DnaB onto diverse replication fork substrates.

**Figure 3:**
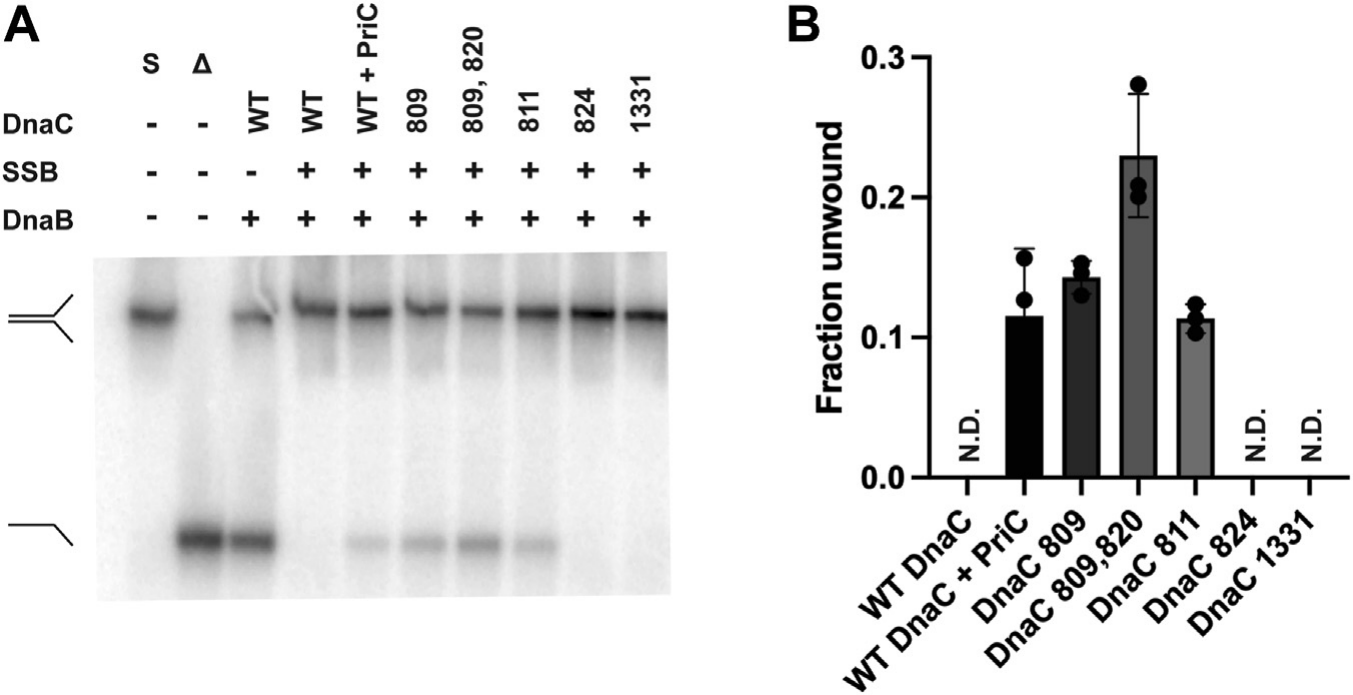
**DnaC variants load DnaB onto an SSB-coated “simple” replication fork.***A*, helicase loading assay with replication fork described in Figure 2. The formation of unwound products indicates DnaB has been loaded and unwinds the parental duplex. Unwinding was measured three times, with the gel representative of multiple experiments. S = substrate and Δ = boiled substrate (expected product). *B*, bars display the mean fraction DNA unwound from three independent experiments, with the values for each measurement shown as points. Error bars represent standard error. N.D. = no product detected.

We first examined DnaC-mediated DnaB loading and unwinding of a replication fork with SSB bound to ssDNA leading and lagging strands (Fig. 3). SSB blocked wild-type DnaC-mediated DnaB loading and unwinding of this simple replication fork as expected (Fig. 3*A*, compare lanes 3 and 4, and 3B). As a positive control, PriC, an RRP that loads DnaB onto replication forks with ssDNA ends (29, 31), was included in the assay, resulting in DnaB loading in the presence of SSB and unwinding of ∼10% of the substrate (Fig. 3*A*, lane 5, and *B*).

The DnaB-loading activity of each of the DnaC variants was examined to determine if they had gained the ability to load DnaB onto the SSB-coated simple fork. DnaC 809 efficiently loaded DnaB onto the fork structure in the presence of SSB (Fig. 3*A*, lane 6, and *B*). This result is similar to that observed in a prior study (24). Interestingly, DnaC 809,820, and DnaC 811 were also able to load DnaB onto an SSB-coated simple fork at levels similar to that measured with DnaC 809 (Fig. 3*A*, lanes 7 and 8, and *B*). Neither DnaC 824 nor DnaC 1331 were capable of loading DnaB onto an SSB-coated simple fork, suggesting these variants do not have the same activity as the other three variants. The lack of activity for DnaC 824 contrasts with its bypass activity in cells, suggesting that the *in vitro* reloading assay does not fully recapitulate DnaC 824 action in cells or that the bypass mechanism used by DnaC 824 differs from other DnaC variants. DnaC 1331, which is not a bypass variant, was not expected to load DnaB in the presence of SSB.

**Figure 4:**
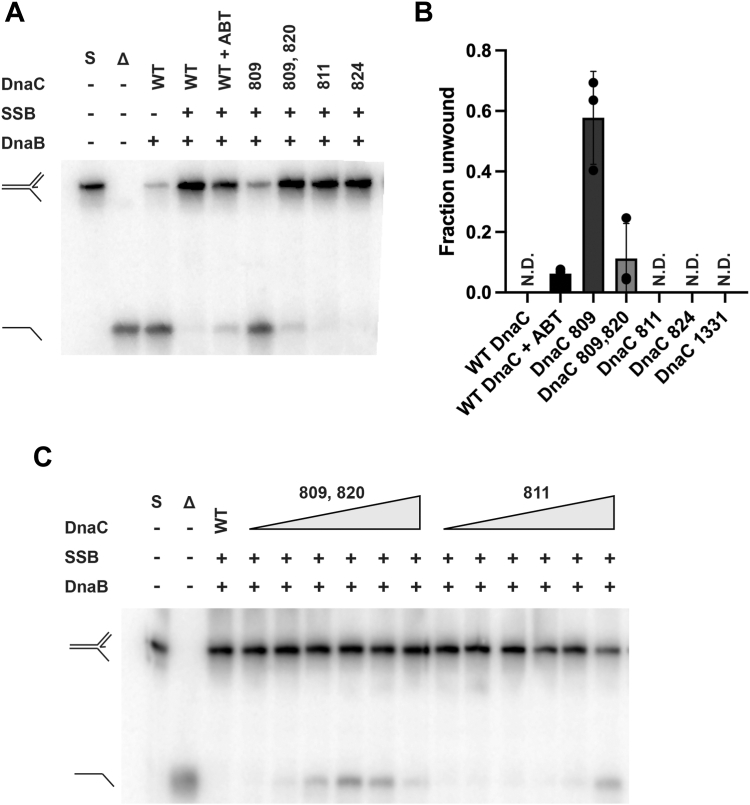
**DnaC variants load DnaB onto SSB-coated replication fork with a nascent leading strand.***A*, helicase loading assay with a replication fork containing 60 bp duplex parental DNA, a 38 base ssDNA lagging strand arm, and a 38 base leading strand arm with a 33 base annealed nascent leading strand (leaving a 5 base ssDNA gap at the fork junction). The formation of unwound product indicates DnaB has been loaded and unwinds the parental duplex. Unwinding was measured three times, with the gel representative of multiple experiments. S = substrate and Δ = boiled substrate (expected product). *B*, bars display the mean fraction DNA unwound from three independent experiments, with the values for each measurement shown as points. Error bars represent standard error. N.D. = no product detected. *C*, titrations of DnaC 809,820 (0, 50, 100, 200, 400, 800 nM) and DnaC 811 (0, 50, 100, 200, 400, 800 nM) in the reconstituted reaction. Unwinding was measured three times, with the gel representative of multiple experiments.

Next, we measured DnaC-mediated loading of DnaB onto a fork with a nascent leading strand (Fig. 4, Table S1). This substrate mimics replication forks with unprimed lagging strands and D-loop DNA structures that are formed as intermediates in double-strand DNA break repair. The PriA/PriB replication restart pathway, which relies on PriA, PriB, and DnaT proteins, loads DnaB onto such forks (29, 30). As observed with the simple fork, wild-type DnaC loading of DnaB onto the replication fork was blocked by SSB (Fig. 4*A*, lanes 3 and 4, and *B*). As a positive control, PriA, PriB, and DnaT were included in the assay, resulting in DnaB loading in the presence of SSB and unwinding of ∼10% of the substrate (Fig. 4*A*, lane 5, and *B*). Similar DnaB loading/unwinding levels have been previously reported for the reconstituted PriA/PriB pathway *in vitro* (29, 32).

Interestingly, the DnaC 809 and DnaC 809,820 bypass variants were able to load DnaB onto this replication fork without the assistance of RRPs (Fig. 4*A*, lanes 6 and 7, and *B*). However, DnaC 809,820 loaded DnaB less efficiently than DnaC 809. Titrations of variants were performed to test whether the two proteins loaded DnaB at different optimal DnaC:DnaB ratios. DnaC 809,820 was more efficient at loading DnaB at DnaC concentrations up to 400 nM. At the highest concentration tested (800 nM), DnaC 809,820 loading of DnaB is less efficient (Fig. 4*C*). DnaC 811 was also able to load DnaB, but only at the highest concentration tested. Wild-type DnaC, DnaC 824, and DnaC 1331 did not load DnaB onto SSB-coated forks with a nascent leading strand under any conditions tested (Fig. S3).

### DnaC 809 loads DnaB onto replication forks with dsDNA lagging strands

**Figure 5:**
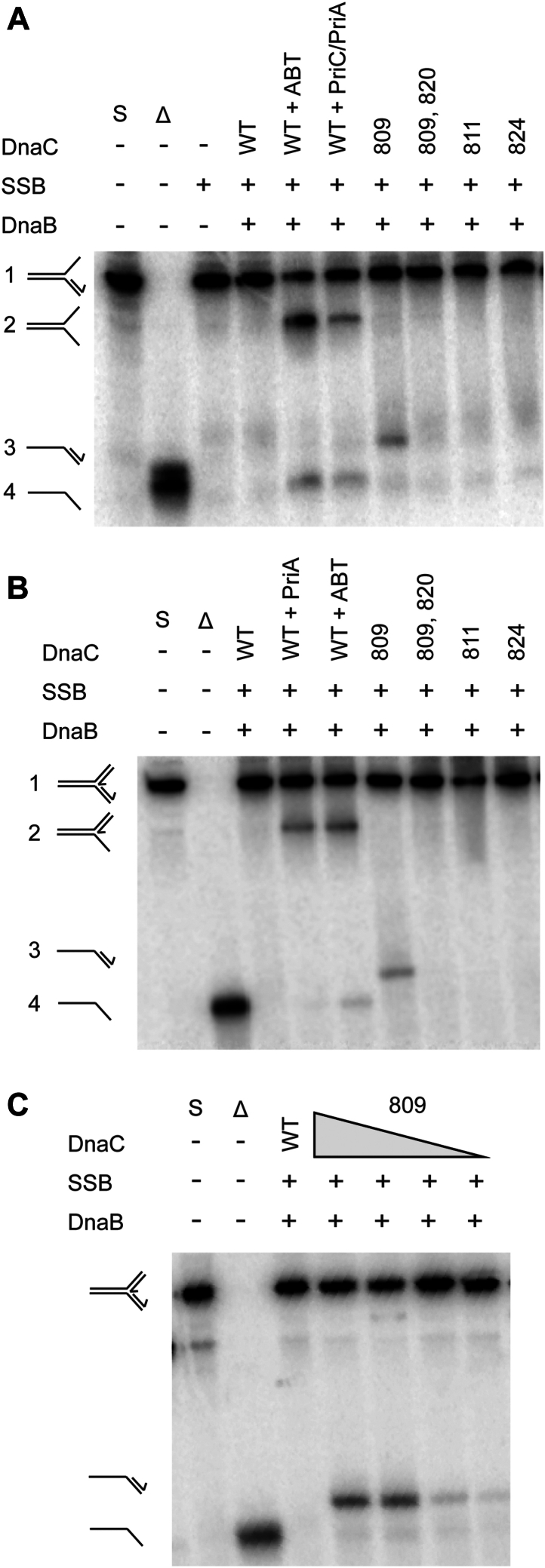
**DnaC 809 loads DnaB onto dsDNA.***A*, helicase loading assay with a replication fork containing 60 bp duplex parental DNA, a 38 base ssDNA leading strand arm, and a 38 bp lagging strand duplex arm. For positive controls, PriA unwinds the nascent lagging strand of the substrate (fork 1) to form fork 2. PriA, PriB, DnaT or PriC then load DnaB onto fork 2 to form fork 4. Fork 3 is formed when DnaC 809 loads DnaB. No helicase is present to unwind the nascent lagging strand, so it remains duplex. S = substrate and Δ = boiled substrate. *B*, helicase loading assay with a replication fork containing a 60 bp duplex parental DNA, a 38 bp duplex lagging strand arm, and a 38 base leading strand arm with a 33 base annealed nascent leading strand (leaving a 5 base ssDNA gap at the fork junction). For positive controls, PriA unwinds the nascent lagging strand of fork 1 to form fork 2. When PriB and DnaT are also added, DnaB is loaded onto fork 2 to form product 4. *C*, titrations of DnaC 809 (0, 50, 100, 200, 400, 800 nM) with the replication fork from (B). Unwinding was measured three times, with the gels representative of multiple experiments.

We next assessed the ability of each DnaC variant to load DnaB onto a synthetic DNA fork that contains a nascent lagging strand. DnaC loads DnaB onto single-stranded lagging strand DNA, positioning DnaB to unwind parental DNA in a 5′ to 3′ direction. To overcome duplex lagging strands in the PriA/PriB replication restart pathway, PriA helicase first unwinds the lagging strand to prepare ssDNA for DnaB loading. PriA then acts with PriB and DnaT to mediate DnaC-dependent DnaB loading onto the replication fork (16). As expected, wild-type DnaC failed to load DnaB onto this replication fork on its own as evidenced by the absence of DnaB-dependent unwinding (Fig. 5*A*, lane 4). However, the inclusion of PriA, PriB, and DnaT led to the unwinding of the lagging strand by PriA and the unwinding of the parental DNA by DnaB (Fig. 5*A*, lane 5). Additionally, the inclusion of PriA and PriC allows for PriA unwinding of the lagging strand followed by PriC-mediated DnaB loading, as reported previously (33) (Fig. 5*A*, lane 6).

Substitution of the DnaC variants in the assay showed that, unexpectedly, DnaC 809 loads DnaB onto the replication fork in the absence of RRPs (Fig. 5*A*, lane 7). Parental duplex unwinding occurred without lagging strand unwinding, indicating that DnaC 809 loaded DnaB onto duplex DNA. None of the other DnaC variants were able to load DnaB onto the fork with a nascent lagging strand.

Finally, we assessed the ability of each variant to load DnaB onto a four-stranded replication fork, with both nascent leading and lagging strands. Wild-type DnaC required PriA, PriB, and DnaT to load DnaB onto the four-stranded fork (Fig. 5*B*, lanes 3 and 5). PriA alone was able to unwind the nascent lagging strand as expected, but this activity was insufficient for loading DnaB by wild-type DnaC (Fig. 5*B*, lane 4). Consistent with the result observed with the previous replication fork, DnaC 809 was able to load DnaB onto the four-stranded replication fork in the absence of all RRPs, leading to unwinding of the parental duplex without lagging strand DNA winding. This further supports to notion that DnaC 809 can bypass the requirement for an ssDNA lagging strand for DnaB loading, loading the helicase directly onto duplex lagging-strand DNA. At higher DnaC 809:DnaB ratios, DnaC 809 loads DnaB onto the four-stranded replication fork even more efficiently (Fig. 5*C*). None of the other DnaC variants were capable of loading DnaB onto the four-stranded replication fork (Fig. 5*B*).

### DnaC 1331 blocks PriA/PriB pathway DnaB loading

**Figure 6:**
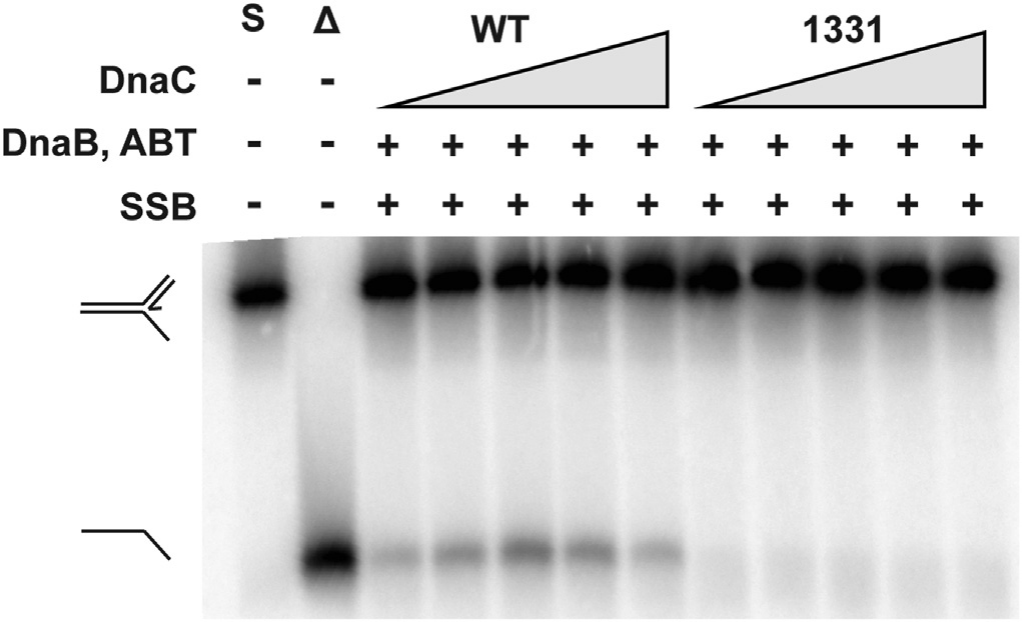
**DnaC 1331 blocks DnaB loading through the PriA/B pathway.** Helicase loading assay with a fork containing 60 bp duplex parental DNA, a 38 base ssDNA lagging strand arm, and a 38 base leading strand arm with a 33 base annealed nascent leading strand (leaving a 5 base ssDNA gap at the fork junction). Formation of unwound product indicates DnaB has been loaded and unwinds the parental duplex. S = substrate and Δ = boiled substrate. DnaC and DnaC 1331 were titrated (50, 100, 200, 400, 800 nM). “ABT” indicates that PriA, PriB and DnaT have been included. Unwinding was measured three times, with the gel representative of multiple experiments.

DnaC 1331 is unique among the DnaC variants in this study in that it is not an RRP deficiency suppressor. Instead, *dnaC1331* phenocopies Δ*priB in vivo*, suggesting that DnaC 1331 could block the PriA/PriB replication restart pathway (22). DnaC 1331 can support DnaB loading onto ssDNA in the absence of SSB but not when SSB is included (Figs. 2 and 3), which parallels wild-type DnaC activity. To test whether DnaC 1331 alters replication restart, we measured the ability of DnaC 1331 to load DnaB onto an SSB-bound replication fork *via* the PriA/PriB pathway. With wild-type DnaC in this assay, DnaB loading and unwinding are observed when PriA, PriB, and DnaT are present (Fig. 6, lanes 3–7). However, substituting DnaC 1331 in the assay blocks DnaB loading and unwinding is not observed (Fig. 6, lanes 8–12). Thus, DnaC 1331 impairs loading by the PriA/PriB restart assay *in vitro*, which parallels *dnaC1331* acting as a Δ*priB* phenocopy *in vivo* (25).

## Discussion

DnaC loads the replicative helicase, DnaB, onto DNA to initiate replication and replication restart. To better understand how mutations of *dnaC* impact replication and replication restart, we purified a panel of DnaC variants with altered cellular functions and tested their DnaB loading abilities *in vitro*. Four of the DnaC variants act as suppressors that can bypass normal DNA replication restart processes in cells whereas a fifth phenocopies deletion of the *priB* replication restart gene. All five DnaC variants were able to load DnaB onto ssDNA in the absence of SSB. Interestingly, three of the bypass variants, DnaC 809, DnaC 809,820 and DnaC 811, were also able to load DnaB onto SSB-coated replication forks in a reconstituted helicase loading assay, which represents a gain of function relative to wild-type DnaC. DnaC 809 also had the unexpected ability to load DnaB onto replication forks with a duplex lagging strand. These results point to a loosening of substrate fidelity (and broadening use of diverse replication fork substrates) as an important aspect of the DnaC variants’ ability to bypass DNA replication restart pathways *in vivo.* DnaC 1331 blocks replication restart through the PriA/PriB replication restart pathway, which aligns with the observation that the *dnaC1331* allele phenocopies a Δ*priB* mutation *in vivo*. Taken together, our results suggest that DnaC plays important roles in selecting replication fork substrates onto which DnaB can be loaded and that sequence changes in DnaC can alter coordination with RRPs in a manner that allows bypass or blockage of restart in *E. coli.*

In our analysis, we found that DnaC bypass variants differ in the breadth of their activities, with most loading DnaB onto a wider range of substrates than wild-type DnaC. The results point to possible structural or regulatory impacts of the sequence changes present in the variants. A comparison of DnaC 809 and DnaC 809,820 potentially offers insights into the differences among the DnaC bypass variants. DnaC 811 behaved quite similarly to DnaC 809,820 with respect to the breadth of substrates it can use for DnaB loading, albeit with lower apparent activity levels than DnaC 809,820.

DnaC 809 displayed the broadest activity profile observed among our panel of DnaC variants, loading DnaB onto replication forks with single-stranded, SSB-bound, and duplex lagging-strand DNA. DnaC 809,820 also loads DnaB onto more substrates than wild type DnaC, but it is restricted to ssDNA and SSB/ssDNA lagging strand substrates. *dnaC809* was originally isolated in a *priA*-deficient strain and the mutation suppresses most phenotypes associated with Δ*priA* but it requires PriC and Rep *in vivo* (22, 23). *dnaC809,820* was isolated in a Δ*priB* Δ*priC dnaC809* strain and can suppress lethality caused by deletion of *priA* and *priC* or *rep* (22, 23). The results presented here show that DnaC 809 and DnaC 809,820 can both load DnaB onto replication forks that can be processed by the PriA/PriB and PriC pathways. PriA/PriB is highly active on substrates with little or no ssDNA gap in the nascent leading strand while PriC operates best on substrates that contain long gaps in the nascent leading strand (29). Also, prior studies have shown that DnaC 809 can bypass the need for RRPs to initiate DNA replication on D-loop DNA structures, which have little or no ssDNA leading strand gaps (34). Consistent with these prior studies, our results suggest that most PriA/PriB and PriC replication forks can serve as substrates for DnaB loading by either DnaC 809 or DnaC 809,820 *in vivo.*

The *dnaC809* mutation encodes for an E176G substitution. E176 lies near the ssDNA entry channel on DnaC, and it is adjacent to the side chain of K178 (the residue altered in *dnaC820*) in the DnaC/DnaC interface (Fig. 1) (9). Two highly conserved residues adjacent to the DnaC 809 mutation site, S177 and Y179, are directly involved in binding to ssDNA in the DnaB_6_/DnaC_6_ structure (9). While we did not detect a change in ssDNA binding affinity with DnaC 809, mutations near the ssDNA channel may alter how DnaB_6_/DnaC_6_ loads onto DNA or interacts with various DNA substrates.

The *dnaC820* mutation encodes for a K178N substitution. Although decreasing the positive charge near the ssDNA pore doesn’t appear to impact ssDNA affinity when combined with the *dnaC809* mutation, it could significantly alter the DnaC/DnaC interface (Fig. 1). These differences may contribute to the lower activity of DnaC 809,820 compared to DnaC 809 on some replication fork substrates examined in our study (Figs. 4 and 5). Why *dnaC809,820* suppresses more phenotypes *in vivo* is a mystery. One possibility is that indiscriminate DnaB loading by DnaC 809 is a liability to cell growth whereas the additional mutation in DnaC 809,820 alleviates some promiscuity making it more advantageous *in vivo*. This may also explain why *dnaC809* is dependent upon *priC in vivo* while *dnaC809,820* is not. Our results suggest that DnaC 809,820 may be better able to recognize proper substrates for DnaB loading than DnaC 809.

DnaB loading is typically restricted to ssDNA, loading onto the lagging strand where it then proceeds in the 5′ to 3′ direction to unwind parental duplex DNA (26, 35, 36). Although DnaB loading onto duplex DNA has never before been observed, DnaB has been shown to be able to translocate over duplexes, which requires the DnaB pore to expand to accommodate duplex DNA (26, 27). We have shown that DnaC 809 can load DnaB onto replication forks that have duplex DNA on the lagging strand. How DnaC 809 does this remains unknown. We speculate that the crack in the DnaB_6_/DnaC 809_6_ complex could possibly expand wider than that in wild-type DnaB_6_/DnaC_6_ to accommodate duplex DNA. The E176G mutation may lead to greater flexibility in the DnaB_6_/DnaC_6_ complex, which expands the DNA entry channel for DnaB loading.

DnaC 824 was not found to bypass RRPs in our reconstituted replication restart assays. This was unexpected since the *dnaC824* mutations suppress the phenotypes of Δ*priB priA300* (*priA300* encodes an ATPase-deficient PriA missense variant (25, 37)), Δ*rep priA300*, and Δ*priB* Δ*priC* strains (25, 37)). In the Δ*priB* Δ*priC* context, all three replication restart pathways are inactive. In contrast to the other *dnaC* suppressor mutations, *dnaC824* can only weakly suppress Δ*priA* phenotypes, indicating it retains some dependence upon PriA. However, DnaC 824 remained inactive in our replication restart systems even when PriA was included (and with other replication restart proteins omitted). It is therefore likely that our *in vitro* system does not entirely recapitulate the reactions stimulated by DnaC 824 *in vivo*. Moreover, the distinct behavior of DnaC 824 suggests that while most DnaC bypass variants appear to suppress RRP deficiencies by expanding their activity on diverse DNA replication forks, at least one does not. This argues that there are likely multiple physical mechanisms by which RRP bypass can occur.

Finally, the DnaC 1331 variant blocked restart through the PriA/PriB pathway *in vitro*. DnaC has not been shown to directly interact with PriA, PriB, or DnaT, although DnaB_6_/DnaC_6_ most likely interacts with at least one protein within the system to stimulate DnaB loading. It is possible that the DnaC 1331 mutation (A84Y) directly or indirectly alters a surface that is critical for interaction with one of the RRPs in the PriA/PriB pathway and that disruption of this interface blocks replication restart.

Overall, our results show that variants of DnaC derived from genetic studies offer novel insights into the mechanisms that govern replication fork selection by DnaB_6_/DnaC_6_ and the interplay between DnaB_6_/DnaC_6_ and RRPs. Sequence changes in DnaC confer expanded substrate utilization for DnaB loading, which, in most cases, allows the bypass of RRPs *in vivo* (37). All the mutations described in our study lie within the C-terminal AAA+ domain of DnaC, pointing to an important role for the domain in regulating DnaB_6_/DnaC_6_ interactions with replication fork substrates and RRPs. In addition to offering insights into the general DnaC-mediated DnaB loading process, the DnaC variants described here may prove useful for molecular biology applications in which DnaB loading onto duplex DNA would be beneficial.

## Experimental procedures

### Protein purification

6XHis-MBP tagged *E. coli dnaC* and *dnaC* mutant genes were cloned into pET28b-derived plasmids. Wild-type DnaC was purified as previously described (28). Briefly, DnaC cultures of C43 *E. coli* cells (Lucigen) carrying the DnaC-encoding plasmids were grown by shaking at 37 °C. DnaC variant expression was induced at the mid-log phase (OD_600_ ∼0.4) by the addition of 1 mM isopropyl B-D-1-thiogalactopyranoside (IPTG). After induction, cells were grown for an additional 2.5 h. Cultures were then clarified *via* centrifugation and cell pellets were resuspended in Lysis Buffer supplemented with protease inhibitors (50 mM HEPES-KOH pH 7.5, 1 M KCl, 10% glycerol, 30 mM imidazole, 10 mM MgCl_2_, 0.1 mM ATP, 1 mM β-mercaptoethanol (BME), 2 mM phenylmethylsulfonyl fluoride (PMSF), 2 mM benzamide, half protease inhibitor tablet (Sigma)). Resuspended cells were stored at −80 °C until purification.

Cells were thawed on ice, lysed by sonication, and clarified *via* centrifugation. Clarified lysates were loaded onto a 5 ml HisTrap FF crude FPLC column (GE) equilibrated in Lysis Buffer. The column was washed with 10 ml of Wash Buffer (50 mM HEPES-KOH, pH 7.5, 0.5 M KCl, 10% (v/v) glycerol, 20 mM imidazole, 10 mM MgCl_2_, 0.05 mM ATP, 1 mM BME and protease inhibitors) then DnaC was eluted with 30 ml of Elution Buffer (50 mM HEPES-KOH pH 7.5, 0.5 M KCl, 10% glycerol, 500 mM imidazole, 10 mM MgCl_2_, 0.1 mM ATP, 1 mM BME and protease inhibitors). The eluate was concentrated to 2 ml and applied to a HiPrep S100 FPLC column (GE) equilibrated in 50 mM HEPES-KOH pH 7.5, 500 mM KCl, 10% glycerol, 10 mM MgCl_2_, 0.1 mM ATP, 1 mM BME and protease inhibitors). Peak fractions were concentrated to 1 ml and incubated overnight at 4 °C with Tobacco Etch Virus protease overnight to remove the 6XHis-MBP tag. The cleaved protein was diluted with Dilution Buffer (30 mM Tris-HCl pH 7.5, 10% glycerol, 10 mM MgCl_2_, 0.1 mM ATP, 1 mM BME and protease inhibitors) and loaded onto a HiPrep Q Sepharose fast flow (QFF) FPLC column (GE) equilibrated in QFF buffer (30 mM Tris-HCl pH 7.5, 20 mM KCl, 10% glycerol, 10 mM MgCl_2_, 0.1 mM ATP, 1 mM BME and protease inhibitors). DnaC was eluted with a linear KCl gradient. Eluted protein was concentrated and dialyzed overnight against storage buffer (20 mM HEPES-KOH pH 7.5, 500 mM KCl, and 50% glycerol).

For the DnaC variants, overexpression led to significantly slowed cell growth. To overcome this problem, a strategy was used to target the variants to the periplasm. Wild-type *dnaC* was cloned as a 6XHis-MBP fusion with an N-terminal periplasmic localization signal and the *dnaC* point mutations were introduced *via* site-directed mutagenesis. All constructs were sequenced to verify the mutations. Variants were expressed in arabinose-inducible BL21 cells by induction at mid-log phase (OD_600_ ∼0.6) with 0.5 mM IPTG and 0.1% arabinose overnight at 16 °C. Purification followed the wild-type DnaC protocol, except that an additional maltose affinity gravity column was used to further separate MBP from DnaC variants following ion exchange purification.

*E. coli* wild-type DnaB, PriA, PriB, PriC, SSB, and DnaT were purified as previously described (32, 38).

### Construction of synthetic replication fork substrates

Synthetic DNA replication forks were generated as previously described (29). Briefly, oligonucleotide 3L-98 was 5′-radiolabeled with ATP-[γ-^32^P] using T4 polynucleotide kinase and annealed to the complementary strand (1b-98) with or without nascent leading (11b-38) and lagging strands (b-33) (Table S1). Forks were purified *via* PAGE electrophoresis. The resulting substrates have a 60-bp parental duplex, with either a 38-base ssDNA or a 38-bp leading strand duplex DNA, and either a 38-base ssDNA or a 33-base pair lagging strand duplex DNA with a 5-base ssDNA gap at the fork junction.

### Analytical size exclusion chromatography

10 nmol of DnaB and 12 nmol of DnaC were diluted into 5 ml of S6 Buffer (20 mM Tris-HCl pH 8.5, 0.2 M NaCl, 5 mM MgCl_2_, 1 mM BME, 1 mM ATP) and concentrated to 0.5 ml. Samples were loaded onto a Superdex 600 Increase 10/300 Gl analytical size exclusion FPLC column (Cytiva) and eluted with S6 buffer. Peak fractions were analyzed *via* PAGE (4–15% gel) for the presence of DnaB and DnaC.

### DnaC ssDNA binding assays

Fluorescence anisotropy assays were carried out as previously described (9) with minor modifications. DnaC (0–50 μM) was diluted into 50 mM HEPES-KOH, pH 7.5, 125 mM KCl, 10% glycerol, 5 mM MgCl_2_, 2 mM ATP, 2 mM DTT, 0.1 mg/ml BSA and 10 nM fluorescein-labeled dT_20_ oligonucleotide. Samples were incubated at 37 °C for 15 min then fluorescence anisotropy values were recorded at 25 °C using a Beacon 2000 fluorescence polarization system. Fluorescence anisotropy values were normalized to a no-DnaC control. Data points are the mean from three independent measurements, with error bars representing standard error. Due to limitations in the highest concentrations of DnaC and DnaC variants that could be tested, binding saturation was not reached, and interpretations were restricted to qualitative comparisons.

### DnaB loading assay

DnaB loading assays were carried out as previously described (29). Briefly, 1 nM (molecules) synthetic DNA replication fork was incubated with either 0 nM or 60 nM SSB (simple fork) or 120 nM SSB (leading, lagging, full forks) in reaction buffer (50 mM HEPES pH 7.0, 0.04 mg/ml BSA, 2 mM DTT, 2 mM ATP, 4 mM Magnesium acetate). Loading reactions were initiated by the addition of 240 nM DnaB and 200 nM DnaC variants unless otherwise indicated. Where indicated, 10 nM PriA, 100 nM PriB, and 960 nM DnaT or 40 nM of PriC were also added. The reactions were incubated at 37 °C for 30 min and terminated with 20 mM EDTA, 0.5% SDS, and 0.2 g/L protease K. DNA loading buffer was added, and the samples were resolved by electrophoresis using a 10% TBE-PAGE gel. Gels were quantified using ImageQuant and analyzed with the GraphPad Prism software. Data in graphs are represented as fractions unwound from three independent experiments, with the value for each independent measurement shown. Error bars represent standard error.

## Data availability

All data described in this study are presented in the article and accompanying supporting information. All original data are freely available through the Dryad repository (https://doi.org/10.5061/dryad.qrfj6q5pg).

## Supporting information

This article contains supporting information (33).

## Conflict of interest

The authors declare that they have no conflicts of interest with the contents of this article.
