# Supplementary material for: Biochemical characterization of Escherichia coli DnaC variants that alter DnaB helicase loading onto DNA
Source: J Biol Chem. 2024 Apr 6;300(5):107275. doi: 10.1016/j.jbc.2024.107275 (PMC11087952; doi:10.1016/j.jbc.2024.107275)
Supplement: Supporting Information [file mmc1.pdf]

Supporting information for:

Biochemical characterization of *E. coli* DnaC variants that alter DnaB helicase loading onto DNA

Sarah D. McMillan and James L. Keck

Table S1

Figures S1-S3

Table S1: Oligonucleotides used to make synthetic replication forks.

Name	Sequence (5′-3′)	Substrate name	Source
1b-98	GCA AGC CTT CTA CAG GTC GAC CGT CCA TGG CGA CTC GAG ACC GCA ATA CGG ATA AGG GCT GAG CAC GCC GAC GAA CAT TCA CCA CGC CAG ACC ACG TA	Leading parental strand	(Heller and Marians, 2005b)
3L-98	GAC TAT CTA CGT CCG AGG CTC GCG CCG CAG ACT CAT TTA GCC CTT ATC CGT ATT GCG GTC TCG AGT CGC CAT GGA CGG TCG ACC TGT AGA AGG CTT GC	Lagging parental strand	(Heller and Marians, 2005b)
11b-38	TAC GTG GTC TGG CGT GGT GAA TGT TCG TCG GCG TGC TC	Leading nascent strand	(Heller and Marians, 2005b)
b-33	AGT CTG CGG CGC GAG CCT CGG ACG TAG ATA GTC	Lagging nascent strand	(Heller and Marians, 2005b)

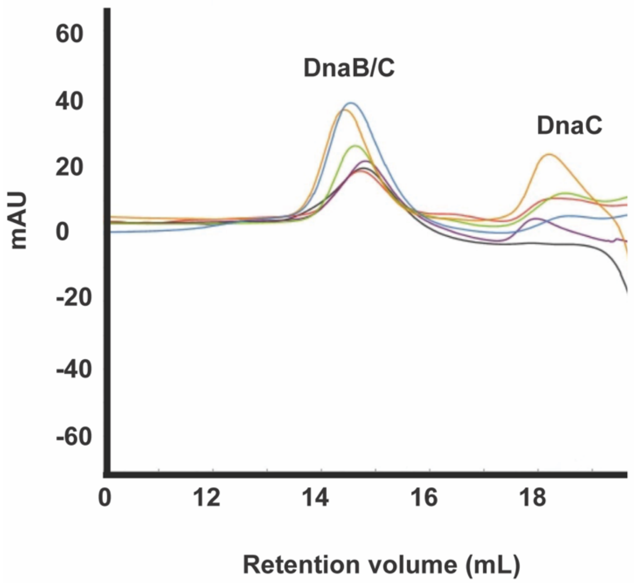

Figure S1: DnaC variants co-elute with DnaB through size exclusion chromatography. FPLC traces show wild-type DnaC (blue), DnaC 809 (black), DnaC 809,820 (orange), DnaC 811 (purple), DnaC 824 (red), or DnaC 1331 (green) co-elution with DnaB. DnaC alone elutes at 19 mL in this experiment.

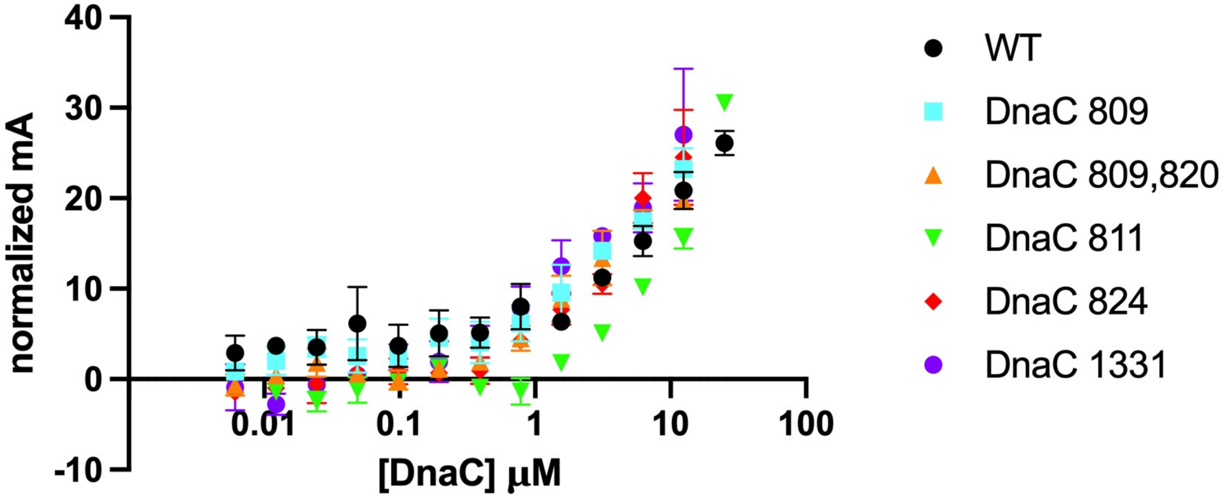

Figure S2: DnaC variants retain ssDNA binding. Fluorescence anisotropy assays show that wild-type DnaC (black), DnaC 809 (blue), DnaC 809,820 (orange), DnaC 811 (green), DnaC 824 (red), or DnaC 1331 (purple) retain the ability to bind ssDNA. Data points are the mean from three independent measurements, with error bars representing standard error.

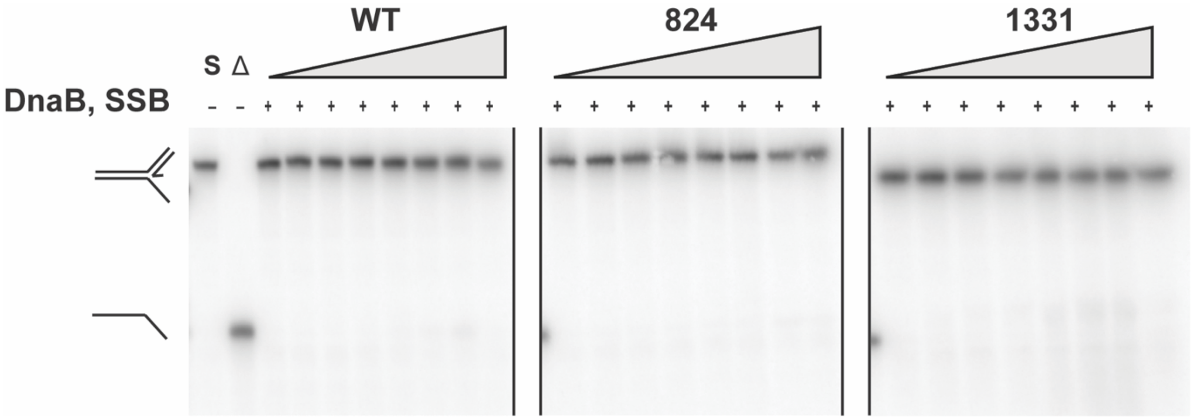

Figure S3: Wild type DnaC, DnaC 824 and DnaC 1331 do not load DnaB onto an SSB-coated fork with a nascent leading strand. Helicase loading assay with a fork containing 60 bp duplex parental DNA, a 38 base ssDNA lagging strand arm, and a 38 base leading strand arm with a 33 base annealed nascent leading strand (leaving a 5 base ssDNA gap at the fork junction). Formation of unwound product would indicate DnaB has been loaded and unwinds the parental duplex; this is not observed. Titrations of each DnaC variant were performed at 0, 50, 100, 200, 400, 800 1600 or 3200 nM.
